# Up-Regulation of the Nrf2/HO-1 Antioxidant Pathway in Macrophages by an Extract from a New Halophilic Archaea Isolated in Odiel Saltworks

**DOI:** 10.3390/antiox12051080

**Published:** 2023-05-11

**Authors:** Javier Ávila-Román, Patricia Gómez-Villegas, Carla C. C. R. de Carvalho, Javier Vigara, Virginia Motilva, Rosa León, Elena Talero

**Affiliations:** 1 Department of Pharmacology, Faculty of Pharmacy, Universidad de Sevilla, Profesor García González Street, 41012 Seville, Spain; 2 Laboratory of Biochemistry, Center for Natural Resources, Health, and Environment, Universidad de Huelva, Avda. de las Fuerzas Armadas s/n, 21071 Huelva, Spain; 3 iBB—Institute for Bioengineering and Biosciences, Department of Bioengineering, Instituto Superior Técnico, Universidade de Lisboa, Av. Rovisco Pais, 1049-001 Lisbon, Portugal; 4 Associate Laboratory i4HB—Institute for Health and Bioeconomy, Instituto Superior Técnico, Universidade de Lisboa, Av. Rovisco Pais, 1049-001 Lisbon, Portugal

**Keywords:** bacterioruberin, carotenoids, fatty acids, haloarchaea, inflammation, macrophages, Odiel Saltworks, oxidative stress

## Abstract

The production of reactive oxygen species (ROS) plays an important role in the progression of many inflammatory diseases. The search for antioxidants with the ability for scavenging free radicals from the body cells that reduce oxidative damage is essential to prevent and treat these pathologies. Haloarchaea are extremely halophilic microorganisms that inhabit hypersaline environments, such as saltworks or salt lakes, where they have to tolerate high salinity, and elevated ultraviolet (UV) and infrared radiations. To cope with these extreme conditions, haloarchaea have developed singular mechanisms to maintain an osmotic balance with the medium, and are endowed with unique compounds, not found in other species, with bioactive properties that have not been fully explored. This study aims to assess the potential of haloarchaea as a new source of natural antioxidant and anti-inflammatory agents. A carotenoid-producing haloarchaea was isolated from Odiel Saltworks (OS) and identified on the basis of its 16S rRNA coding gene sequence as a new strain belonging to the genus *Haloarcula*. The *Haloarcula* sp. OS acetone extract (HAE) obtained from the biomass contained bacterioruberin and mainly C18 fatty acids, and showed potent antioxidant capacity using ABTS assay. This study further demonstrates, for the first time, that pretreatment with HAE of lipopolysaccharide (LPS)-stimulated macrophages results in a reduction in ROS production, a decrease in the pro-inflammatory cytokines TNF-α and IL-6 levels, and up-regulation of the factor Nrf2 and its target gene heme oxygenase-1 (HO-1), supporting the potential of the HAE as a therapeutic agent in the treatment of oxidative stress-related inflammatory diseases.

## 1. Introduction

Many pathologies such as inflammatory bowel disease, psoriasis, and rheumatoid arthritis have an inflammatory base, triggered in part by a deficient antioxidant system that leads to ROS overproduction and chronic inflammation. Impairment of the antioxidant defense systems causes the overaccumulation of these oxidative species, which produces oxidative damage in lipids, proteins, and DNA, inducing cell death [1]. For this reason, new sources of antioxidants are desirable, not only to treat chronic diseases or promote health but also to be used as additives for food and pharmaceutical products, in order to prevent their deterioration and extend their shelf-life. Most natural antioxidants ingested by humans come from plants; however, microorganisms can also be a source of these compounds to substitute undesirable artificial ones [2,3]. In this regard, extremely halophilic microorganisms that inhabit hypersaline environments, such as saltworks or salt lakes, are particularly interesting due to their ability to cope with extreme conditions. High salinity is the main hindrance found in these habitats, but other extreme conditions, including high ultraviolet (UV) and infrared radiations from the sun, must also be endured. Halophilic archaea are usually the main representatives of the microbiota at the highest salinity (≥5 M NaCl), exceeding Bacteria and Eukarya [4,5]. Haloarchaea possess specific strategies to proliferate in such harsh environments, which include the accumulation of isomolar concentrations of KCl to balance the osmotic pressure with the surrounding medium, and an adapted cellular machinery able to work at high salt concentrations [6]. In addition, Archaea count with unique cell membranes, which contain isoprenoids ether linked to *sn*-glycerol-1-phosphate, contrarily to bacterial and eukaryotic cells where the fatty acid chains are ester linked to the *sn*-glycerol-3-phosphate backbone. This lipid structure is totally different from that found in the membrane of bacteria or eukaryotes and is one of the structural characteristics that supported the definition of Archaea as an independent domain of life [7]. Previous studies have shown that several archaeal species contain fatty acids (FAs) and phospholipid FAs [8,9,10,11]; nonetheless, there is practically no information about the physiological role and the composition of haloarchaeal FAs.

Most members of the class Halobacteria are pink or red pigmented due to the presence of carotenoids and retinal proteins in their membranes [12]. Carotenoids are one of the most abundant bioactive compounds in the marine environment and are highly demanded products with many applications in cosmetics, pharmaceutics, nutraceuticals, foods, and beverages [13]. These pigments are usually obtained from plants, algae, or yeasts, while prokaryotic microbial sources are less exploited. Interestingly, carotenoids obtained from different species of haloarchaea have been reported to exhibit health-promoting properties such as antimicrobial, antihemolytic, neuroprotective, or antiviral, among others [14,15,16,17]. The main carotenoid found in haloarchaea is bacterioruberin, a 50-carbon carotenoid with 13 conjugated double bonds and 4 terminal hydroxyl groups, which give the cells exceptional properties against oxidative stressors, acting as a radical scavenger in hypersaline environments [18]. Bacterioruberin protects the haloarchaea against UV light and could act as a membrane stabilizer, connecting the inner and outer leaflets of the membrane bilayer by the interaction of its hydroxyl groups through hydrogen bonds with the hydrophilic head groups of polar lipids [19]. 

In the present study, a new strain of haloarchaea belonging to the genus *Haloarcula* was isolated from Odiel Saltworks, and the antioxidant and anti-inflammatory properties of an acetone extract from this microorganism, containing carotenoids and FA, were evaluated by both in vitro and cellular assays, to explore the molecular mechanisms underlying these activities. 

## 2. Materials and Methods

### 2.1. Sample Collection and Strain Isolation

Haloarchaeal strains were isolated from water samples from a crystallizer pond in Odiel Saltworks (37.255025, −6.972945), located in the Odiel River Marshlands in Huelva (SW, Spain). Water samples (20 µL) were plated in agar plates and incubated at 37 °C for 30 days. The culture medium used for the isolation contained per liter: 10 g glucose, 156 g NaCl, 13 g MgCl_2_∙6H_2_O, 20 g MgSO_4_∙7H_2_O, 1 g CaCl_2_∙6H_2_O, 4 g KCl, 0.2 g NaHCO_3_, and 5 g yeast extract. The colony with the most intense red color was purified by five streaking rounds on fresh agar plates and preserved in 20% glycerol (*w*/*v*) at −80 °C for further use.

### 2.2. Molecular Identification

The selected microorganism was identified by 16S ribosomal RNA gene sequencing. Genome DNA was extracted by using the Gene JET Genomic Purification kit (Thermo Fisher Scientific, Waltham, MA, USA), following the manufacturer’s instructions. The full length of the 16S rRNA coding gene was amplified with specific primers for archaea 21F (5′-TTCCGGTTGATCCTGCCGGA-3′) and 1492R (5′-GGTTACCTTGTTACGACTT-3′). Polymerase chain reaction (PCR) was carried out in a total volume of 25 µL, using an Eppendorf thermo-cycler. The reaction mixture contained 1 µL of genomic DNA, 0.2 U RED Taq^®^ DNA polymerase (Sigma Aldrich, St. Louis, MO, USA), and 2.5 µL of its specific 10× buffer that contained 10 pM of each primer, 0.2 mM dNTPs, and 2.5 mM MgCl_2_. The thermal profile was set to 0.5 min at 96 °C, 0.5 min at 56 °C, and 1 min at 72 °C for 30 cycles, followed by 10 min of final extension. The PCR products were checked by electrophoresis, on a 1% agarose gel, and sent to Stabvida (Lisbon, Portugal) for Sanger sequencing. The 1.4 kb 16S rRNA gene sequences obtained were compared to those available at the GenBank and the European Molecular Biology Laboratory (EMBL) databases, using the Basic Local Alignment Search Tool (BLASTn) at the National Center for Biotechnology Information (NCBI) [20]. 

### 2.3. Culture Medium and Extract Preparation

The selected microorganism was grown in the liquid medium previously detailed for the strain isolation at 37 °C and 150 rpm for 10 days, and the growth was monitored according to the optical density at 580 nm. When the culture reached the stationary phase of growth, 1 L was collected by centrifugation at 19,800× *g* for 15 min at 4 °C. The cellular pellet was treated overnight with 10 mL of cold acetone (−20 °C) per gram of fresh biomass in the dark, and vortex several times until a colorless pellet was obtained. Then, the sample was centrifuged at 4 °C and 19,800× *g* for 30 min, and the supernatant, which contains carotenoids and other lipophilic compounds, was dried in a rotary vacuum evaporator and freeze dried. 

### 2.4. Lipid Analysis

The FAs were extracted and simultaneously methylated to fatty acid methyl esters (FAMEs) using the Instant FAME^TM^ kit from MIDI, Inc. (Newark, DE, USA), after acetone removal at 40 °C and 100 mBar on a vacuum evaporation system (RapidVap from Labconco, Kansas City, MO, USA). At least two samples of extract were used per lipid extraction. For lipid quantification, the FA C19:0 (Sigma-Aldrich, Darmstadt, Germany), was added as an internal standard prior to transesterification. The base-catalyzed kit allows the methylation of FA to FAMEs and their extraction to an organic solvent for analysis. 

The FAMEs were analyzed by gas chromatography on a 6890N gas chromatograph, equipped with a flame ionization detector (FID) and a 7683B series injector, all from Agilent Technologies (Santa Clara, CA, USA). The gas chromatograph was equipped with a 25 m long Agilent J&W Ultra 2 capillary column and was programmed and controlled by the Sherlock software package, version 6.2 (from MIDI, Inc.). Peak identification was carried out by Sherlock Software by comparison with standards. Their identification was confirmed by injection of the same samples on an Agilent 7820A gas chromatograph equipped with a 7693A autoinjector and a 5977E quadrupole mass spectrometer (MS; Agilent Technologies, Santa Clara, CA, USA). A 25 m long Agilent J&W Ultra 2 capillary column was used, and the carrier gas was helium at 1.5 mL/min.

### 2.5. Carotenoid Analysis

The content of total carotenoids in the acetone extract was calculated by the absorbance of the sample at 496 nm in a cuvette with a 1 cm path length and using the specific absorption coefficient A^1%^ = 2540 (100 mL g^−1^ cm^−1^) [21]. The chromatographic analysis of the carotenoids present in the extract was performed in a Merck-Hitachi LaChrom Elite (Prague, The Czech Republic) HPLC equipped with a DAD (L-2455) detector. Separation of carotenoids was performed using an RP-18 column (5 µm × 25 cm × 4.6 mm id), with an equilibration time of 20 min, and a flow rate of 1 mL min^−1^. Solvents were (A) a mixture of acetonitrile/water (9:1 *v*/*v*) and (B) pure ethyl acetate. The gradient elution program was as follows: 0–16 min 0–60% A; 16–30 min 60% A; 30–35 min 100% A. The column temperature was kept at 25 °C, and the injection volume was 100 µL. Chromatograms were recorded in the range of 450 nm and the EZ ChromeElite program was used for data processing. The compounds were identified on the basis of their retention time and UV–Vis spectra. 

### 2.6. Antioxidant Capacity Assessment

The antioxidant activity of the compounds as a scavenger was performed by the ABTS colorimetric method [22]. The ABTS·+ radical was generated with 3.84 mg/mL of ABTS (Applichem, Darmstadt) and 6.6 mg/mL of potassium persulfate (Sigma-Aldrich Química, SA, Spain) dissolved in water and incubated for at least 16 h in the dark. The compounds were prepared into 96-well plates at different concentrations (0–200 μg/mL) and ABTS·+ solution, equivalent to an absorbance of 0.7 ± 0.02, was incubated with samples for 6 min at room temperature. Then, the absorbance was measured at 734 nm by using a Synergy HT multimode microplate reader (BioTek Instruments, Winooski, VT, USA). Trolox was used as the standard antioxidant in the same concentration range. The effective concentration 50% (EC_50_) was calculated, and results were expressed as Trolox Equivalent Antioxidant Capacity, TEAC (EC_50_ sample/EC_50_ Trolox).

### 2.7. Cell Cultures

Human acute monocytic leukemia cell line, THP-1, was purchased from the American Type Culture Collection (TIB-202, ATCC, Manassas, VA, USA) and cultured in RPMI 1640 media (GIBCO^®^, Life Technologies, New York, NY, USA) containing 10% heat-inactivated fetal bovine serum, 100 U/mL penicillin, and 100 mg/mL streptomycin (PAA^®^, Pasching, Austria), in a humidified atmosphere containing 5% CO_2_ at 37 °C. 

### 2.8. Cell Viability Assay

The in vitro cytotoxicity assessment of HAE was determined by the 3-(4,5-dimethylthiazol-2-yl)-2,5 diphenyltetrazolium bromide (MTT, Calbiochem, Darmstadt, Hesse, Germany) method [23] by using THP-1 cells transformed into macrophages. Briefly, for differentiation into macrophages, THP-1 cells were seeded into 96-well plates (100 μL/well) at a density of 10^5^ cells/mL in the presence of 8 nM phorbol 12-myristate 13-acetate (PMA, Sigma-Aldrich Química, S.A., Madrid, Spain) and, incubated in a humidified atmosphere of 5% CO_2_ at 37 °C for 72 h. Afterward, the medium was removed, and cells were washed twice with ice-cold phosphate saline buffer (PBS, 4 °C). Then, the macrophages were treated with different concentrations of the acetone extract (0, 6.25, 12.5, 25, 50, and 100 µg/mL) for 24 h prepared in DMSO stock solutions at a concentration of 50 mg/mL and diluted to desired concentration directly in the culture medium. Controls were incubated in a fresh medium containing DMSO (0.2% *v*/*v*), which did not affect cell viability. Then, the cells were washed (PBS, 4 °C) prior to the addition of 100 μL of 0.25 mg/mL MTT solution into each well and were incubated for 4 h. The formazan crystals were dissolved with DMSO (200 μL) and 0.1 M glycine buffer pH 10.5 (25 μL). Finally, the absorbance was measured at 550 nm by using a Multiskan EX microplate reader (Labsystems, Thermo Scientific, Helsinki, Finland) and the 50% inhibitory concentration (IC_50_) was calculated.

### 2.9. Intracellular ROS Production

The production of intracellular ROS was determined by using DCF-DA dye assay (ab113851 Abcam, Cambridge, UK) in THP-1 macrophages. Briefly, THP-1 cells were seeded into a 96-well black plate (100 μL/well) at a density of 10^5^ cells/mL with 8 nM PMA as described above. Then, the medium was removed, and cells were washed (PBS, 4 °C). Subsequently, a pretreatment with the HAE (5, 25, and 50 µg/mL), and dexamethasone (Dex) as control (1 µM) was carried out for 1 h. Then, the intracellular ROS production was induced by the addition of lipopolysaccharide from *E. coli* (LPS, 1 µg/mL) for 24 h. Control groups, unstimulated (Control) and stimulated (LPS) were incubated with a medium containing DMSO (0.2% *v*/*v*). Then, the supernatants were removed, and cells were washed (PBS, 4 °C) supplemented with 20 µM DCF-DA (100 µL/well) and incubated for 45 min, according to the manufacturer’s instructions. Fluorescence was measured by using a fluorescence plate reader (Sinergy HT, Biotek^®^, Bad Friedrichshall, Germany) at 485 nm for excitation and 535 nm for emission.

### 2.10. Determination of TNF-α and IL-6 Levels 

The production of pro-inflammatory cytokines was studied in human THP-1 macrophages. Briefly, THP-1 were seeded into a 96-well plate (100 μL/well) at a density of 10^5^ cells/mL in the presence of 8 nM PMA as described previously. After differentiation, the medium was removed, and cells were washed twice (PBS, 4 °C). Subsequently, a pretreatment with the HAE at different concentrations (5, 25, and 50 µg/mL) and Dex (1 µM) as positive reference compound was performed for 1 h. Then, the inflammatory response was induced by the addition of LPS (1 μg/mL) for 24 h. Control groups were incubated with a growth medium containing DMSO (0.2% *v*/*v*). Afterward, the supernatants were collected and stored at −80 °C until cytokines determination by ELISA (Diaclone GEN-PROBE, Besançon, France), according to the manufacturer’s protocol. The absorbance was measured with a microplate reader (Labsystems Multiskan EX, Thermo Scientific, Helsinki, Finland) at 450 nm.

### 2.11. Assessment of Antioxidant Proteins Expression 

The expression of the antioxidant proteins Nrf2 and HO-1 was determined by Western blotting in THP-1 macrophages. Briefly, THP-1 were seeded into 6-well plates (2000 μL/well) at a density of 4 × 10^5^ cells/mL in the presence of 8 nM PMA as previously described. After the incubation period, the medium was removed, the cells were washed twice (PBS, 4 °C), and incubated with different concentrations of the HAE (5, 25, and 50 μg/mL) for 1 h. The inflammatory response was induced by the addition of LPS (1 μg/mL) for 24 h. The Control group was incubated with a growth medium containing DMSO (0.2% *v*/*v*). Subsequently, cell pellets were mixed with cold lysis buffer containing a cocktail of protease inhibitors (50 mM Tris-HCl pH 7.5, 8 mM MgCl_2_, 5 mM ethylene glycol bis (2-aminoethyl ether)-N,N,N’N’-tetraacetic acid, 0.5 mM EDTA, 1 mM phenylmethylsulfonyl fluoride, 0.01 mg/mL leupeptin, 0.01 mg/mL pepstatin, 0.01 mg/mL aprotinin, and 250 mM NaCl (40 µL per sample) and incubated on ice for 30 min. After that, cell homogenates were centrifugated (12,000× *g*, 5 min, 4 °C) to discard cell debris and DNA. The protein cytoplasmic content in the supernatants was determined using the Bradford method. Aliquots of supernatants containing equal protein (50 µg) were separated on 10% acrylamide gel by SDS-polyacrylamide-gel electrophoresis. Then, the proteins were electrophoretically transferred onto a nitrocellulose membrane and incubated with specific primary antibodies as follows: rabbit anti-Nrf2 (1:1000) and rabbit anti-HO-1 (1:1000) (Cell Signaling, Danvers, MA, USA). Each membrane was washed three times for 10 min and incubated with the secondary horseradish peroxidase-linked anti-rabbit (Pierce Chemical, Rockford, IL, USA). To prove equal loading, the blots were analyzed for β-actin expression by using an anti-β-actin antibody (Sigma-Aldrich, St. Louis, MO, USA). Immunodetection was performed by using a chemiluminescence light-detecting kit (Super-Signal West Pico Chemiluminescent Substrate, Pierce, IL, USA). Densitometric data were analyzed following normalization to the housekeeping gene (β-actin), and the signals were quantified with Scientific Imaging Systems (Biophotonics Image J Analysis Software; National Institute of Mental Health, Bethesda, MD, USA).

### 2.12. Statistical Analysis

All values in the figures, tables, and text are expressed as arithmetic means ± standard error of the mean (S.E.M.). Data were evaluated by using the GraphPad Prism Version 6.00 software (GraphPad Software, Inc., San Diego, CA, USA). The Kolmogorov–Smirnov test was used to verify the normality of the data. *p* values < 0.05 were considered statistically significant. The statistical test used for individual analyses is provided in the figure legends.

## 3. Results

### 3.1. Selection and Identification of a Carotenoid-Rich Haloarchaea

A new strain of haloarchaea was isolated from a crystallizer pond in Odiel Saltworks with 32% salinity at the moment of sampling. This microorganism was selected for its fast growth (µ = 1.02 days^−1^), as shown in Supplementary Materials (Figure S1), and intense red color. The identification of the isolate was carried out based on the amplification and sequencing of the complete 16S rRNA encoding gene (Supplementary Materials, Figure S2). The obtained sequence was compared to those available in the NCBI database using the BLASTn tool, showing around 97.5% sequence identity with other *Haloarcula* species, such as *H. hispanica*, *H. japonica*, or *H. marismortui*. In this regard, multiple alignments were generated by Multiple Sequence Comparison by Log-Expectation (MUSCLE). Thus, it was designated as *Haloarcula* sp. OS. 

**Figure 1:**
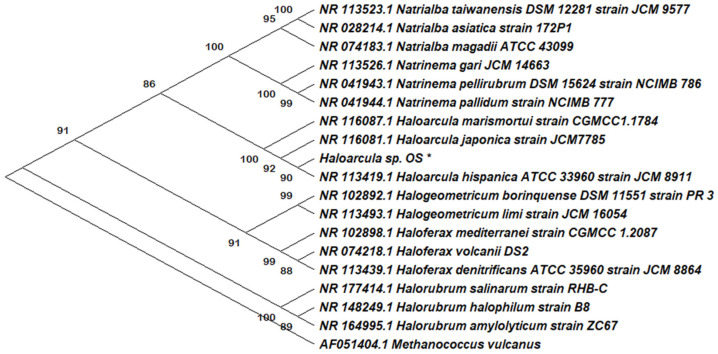
Molecular phylogenetic analysis by the maximum likelihood method. The tree represents a comparison within 16S rRNA coding gene sequences, including a series of reference haloarchaeal species and the new isolate, *Haloarcula* sp. OS, highlighted by an asterisk. The tree was constructed with MEGA X. The numbers at nodes indicate the bootstrap values calculated for 1000 replicates. The name and the NCBI access number are indicated for all the reference sequences. *Methanococcus vulcanus* was used as an outgroup.

A molecular phylogenetic tree was constructed by using the Molecular Evolutionary Genetics Analysis (MEGA X, Version 10, Sudhir Kumar, Pennsylvania State University, US) [24], including the new isolate *Haloarcula* sp. OS and a series of reference haloarchaeal species. The bootstrap was settled on 1000 replicates, and the thermophilic archaea *Methanococcus vulcanus* was set as an outgroup. The 16S rRNA encoding sequence of the new isolate clustered with the corresponding genes of the members of the genus *Haloarcula* (Figure 1).

### 3.2. Characterization of the Haloarcula sp. OS Acetone Extract

**Figure 2:**
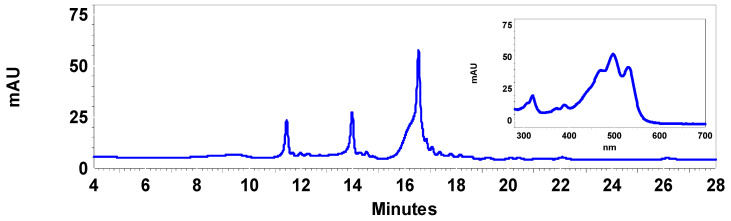
HPLC elution profile of the *Haloarcula* sp. OS acetone extract (HAE). The three prominent peaks observed in the chromatogram correspond to bacterioruberin isomers with the same absorption spectrum, which is shown on the insert. This UV–Vis spectrum with absorption maxima at 468, 496, and 532 nm conforms to the typical three fingers spectrum of bacterioruberin.

An acetone extract was obtained from *Haloarcula* sp. OS (HAE) with the aim of testing its potential as an antioxidant and anti-inflammatory agent. The carotenoid and FA content of this extract was analyzed by RP-HPLC-DAD and GC-MS, respectively. The elution profile of the carotenoids revealed three main peaks with identical absorption spectra with absorption maxima at 468, 496, and 532 nm and two cis absorption maxima at lower wavelengths (Figure 2), which correspond to different bacterioruberin isomers. The concentration of total bacterioruberin, the only carotenoid detected in the HAE, was 10 µg per mg of extract, calculated according to Britton (2004) [21] and using the specific absorption coefficient A^1%^ = 2540 (100 mL g^−1^ cm^−1^).

In addition to carotenoids, other lipid compounds, such as unsaturated FAs, have been shown to play an important role as antioxidants against ROS species. When the HAE was analyzed by gas chromatography-mass spectrometry, significant amounts of 16:0, 18:0, and 18:0 10-methyl FA were identified. This led to the characterization of the extract in terms of FAs. The analysis of the FAs content of the total lipids in the HAE showed that the most abundant FA was 18:0 (55.5% of total lipids), while the others accounted for ca. 0.4–14% (Table 1). The amount of FAs in the extract was 1.7 ± 0.3 µg FA/mg CDW, which corresponds to 0.8 ± 0.06 µg FA/µg extract.

### 3.3. Antioxidant Capacity of the Acetone Extract

**Figure 3:**
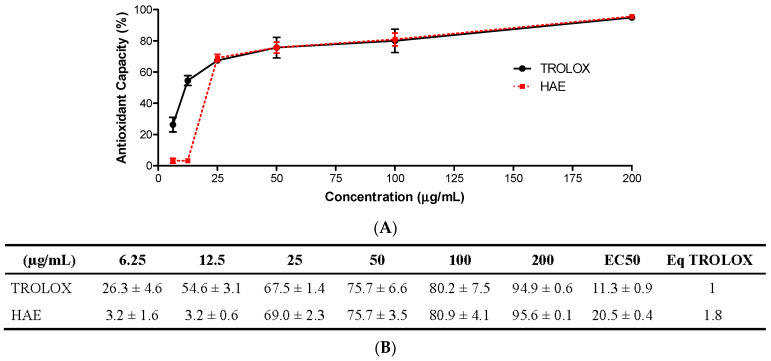
Scavenger antioxidant activity of the *Haloarcula* sp. OS acetone extract (HAE). (**A**) The antioxidant capacity of the HAE at different concentrations (0–200 µg/mL) was determined by ABTS (2,2′-azino-bis (3-ethylbenzothiazoline-6-sulfonic acid)) radical scavenging assay, using TROLOX as a reference control. (**B**) The half-effective concentration (EC_50_) was calculated and the results were expressed as equivalent to Trolox (EC50 sample/EC50 Trolox). Results are representative of three independent experiments (n = 3).

The antioxidant capacity of the HAE was tested using the 2,2′-azino-bis (3-ethylbenzothiazoline-6-sulfonic acid) (ABTS) method. Several dilutions of the HAE were assayed (Figure 3A) to obtain the effective concentration of 50% (EC50), which was expressed as Trolox Equivalent Antioxidant Capacity, TEAC (EC50 sample/EC50 Trolox) (Figure 3B). The reference compound Trolox showed an EC50 of 11.3 ± 0.9 µg/mL. The HAE exhibited a potent antioxidant capacity, displaying an EC50 of 20.5 µg/mL. The HAE showed 1.8 equivalents of Trolox, which means that it is necessary 1.8-fold HAE than Trolox to get the same effect.

### 3.4. Intracellular ROS Assessment

**Figure 4:**
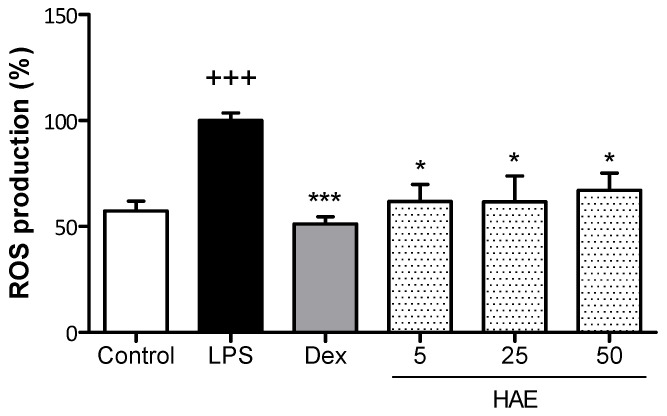
Effect of the *Haloarcula* sp. OS acetone extract (HAE) on the ROS production in LPS-stimulated THP-1 macrophages. THP-1 monocytes were transformed into macrophages with 8 nM PMA for 72 h. Then, cells were pretreated with the BRE (5, 25, and 50 μg/mL) for 1 h and stimulated with LPS (1 µg/mL) for 24 h. Dexamethasone (Dex, 0.39 µg/mL) was used as an internal reference control. Results are representative of four independent experiments (n = 4). Values are the means with standard errors represented by vertical bars. Mean value was significantly different compared to the Control group (+++ *p* < 0.001; Student’s *t*-test). Mean value was significantly different compared to the LPS group (*** *p* < 0.001; * *p* < 0.01; Kruskal–Wallis test).

The levels of intracellular ROS produced in LPS-stimulated THP-1 macrophages were measured by using 2′,7′-dichlorodihydrofluorescein diacetate (DCF-DA) (Figure 4). First, to rule out cytotoxic effects, the HAE was tested at different concentrations (0–100 µg/mL) by using the 3-(4,5-dimethylthiazol-2-yl)-2,5 diphenyltetrazolium bromide (MTT) method. The results showed 100% viability of the macrophages exposed to HAE at all tested concentrations for 24 h (Table 2). Based on these data, the concentrations 5, 25, and 50 μg/mL of HAE were chosen to carry out the ROS assay. 

ROS production was significantly increased in the LPS group in comparison with the Control group (*p* < 0.001), which showed 57.3% of ROS basal levels. The glucocorticoid dexamethasone (Dex, 1 µM) reduced ROS production to similar levels to those of the Control group (*p* < 0.001). The treatment with the HAE caused a significant decrease in ROS production at all tested concentrations (*p* < 0.05), which achieved a reduction close to 40% in ROS levels.

### 3.5. Pro-Inflammatory Cytokines Levels

**Figure 5:**
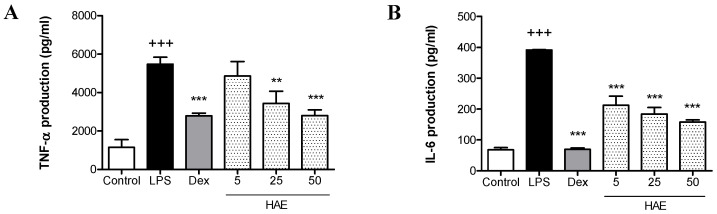
Effect of the *Haloarcula* sp. OS acetone extract (HAE) on the pro-inflammatory cytokines production in LPS-stimulated THP-1 macrophages. THP-1 monocytes were transformed into macrophages with 8 nM PMA for 72 h. Then, cells were pretreated with the HAE (5, 25, and 50 μg/mL) for 1 h and stimulated with LPS (1 µg/mL) for 24 h. Dexamethasone (Dex, 0.39 µg/mL) was used as an internal reference control. (**A**) TNF-α production. (**B**) IL-6 production. Results are representative of five independent experiments (n = 5). Values are the means with standard errors represented by vertical bars. Mean value was significantly different compared with the Control group (+++ *p* < 0.001; Student’s *t*-test). Mean value was significantly different compared with the LPS group (*** *p* < 0.001, ** *p* < 0.01; ANOVA test followed by Tukey’s Multiple Comparison test).

The in vitro anti-inflammatory activity of the HAE obtained from *Haloarcula* sp. OS was evaluated by measuring the production of TNF-α and IL-6 in LPS-stimulated THP-1 macrophages. Our results showed that LPS stimulation induced a marked increase in TNF-α and IL-6 production in THP-1 cells compared to unstimulated control cells (*p* < 0.001). The anti-inflammatory reference drug dexamethasone significantly reduced the levels of both cytokines (*p* < 0.001). As shown in Figure 5A, pretreatment of THP-1 cells with the HAE at the concentrations of 25 and 50 μg/mL resulted in a significant reduction in LPS-mediated TNF-α production (*p* < 0.01 and *p* < 0.001, respectively). Furthermore, the HAE substantially reduced IL-6 levels at all tested concentrations (*p* < 0.001) (Figure 5B).

### 3.6. Assessment of Antioxidant Protein Expression Levels

**Figure 6:**
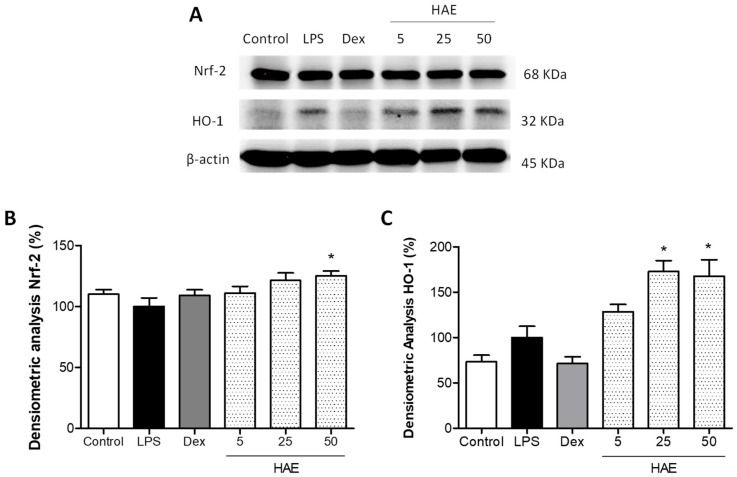
Effect of the *Haloarcula* sp. OS acetone extract (HAE) on the antioxidant Nrf2/HO-1 pathway in LPS-stimulated THP-1 macrophages. THP-1 monocytes were transformed into macrophages with 8 nM PMA for 72 h. Then, cells were pretreated with the HAE (5, 25, and 50 μg/mL) for 1 h and stimulated with LPS (1 µg/mL) for 24 h. Dexamethasone (Dex, 0.39 µg/mL) was used as an internal reference control. (**A**) Representative densitometric analysis of HO-1 and Nrf2 normalized to control (housekeeping gene, β-actin). (**B**) Representative Western blot analysis of Nrf2 and (**C**) HO-1. Results are representative of three independent experiments (n = 3). Values are the means with their standard errors represented by vertical bars. Mean value was compared with the Control group not showing significant differences (LPS vs. Control). Mean value was significantly different compared with the LPS group * *p* < 0.05; Kruskal–Wallis followed by Dunn’s Multiple Comparison test).

To further explore the basis of the potential anti-inflammatory mechanisms of action of the HAE, the expression of the antioxidant proteins Nrf2 and HO-1 was evaluated by Western blotting. THP-1 macrophages were pretreated with the HAE (5, 25, and 50 μg/mL) for 1 h and then stimulated with LPS (1 μg/mL). As presented in Figure 6A, LPS stimulation did not induce significant changes in Nrf2 expression, but a slight non-significant increase in HO-1 expression was observed. However, the pretreatment with HAE resulted in a notable up-regulated expression of the Nrf2 protein at 25 and 50 μg/mL, but only the highest concentration showed a significant increase (*p* < 0.05) in LPS-stimulated macrophages (Figure 6B). This result was correlated with a substantial increase in its target gene HO-1 at concentrations of 25 and 50 µg/mL of the HAE (*p* < 0.05 and *p* < 0.05, respectively) (Figure 6C).

## 4. Discussion

The bioactive properties of haloarchaea extracts are usually linked to the antioxidant capacity of bacterioruberin. The potential of this carotenoid as a radical scavenger has been demonstrated in vitro by several chemical assays (DPPH, ABTS, FRAP, etc.) in different genera of halophilic archaea [15,25,26]. However, the lack of studies on human cell lines makes it difficult to define the pharmacological importance of haloarchaea-derived carotenoids and to extrapolate these results to the development of drugs or health promotion approaches. In this regard, the results obtained in our study have demonstrated, for the first time, the anti-inflammatory properties of HAE in LPS-induced THP-1 macrophages through up-regulation of the Nrf2/HO-1 antioxidant pathway.

Although bacterioruberin is the main carotenoid found in haloarchaea, [27,28,29], the extracts obtained from these microorganisms contain other types of molecules that could potentially have bioactive properties. In this study, we demonstrate that along with bacterioruberin, several membrane FAs are also present in the HAE, being C18:0 the most abundant. It is not always accepted that some archaeal species produce significant amounts of FA, although it has been shown that phospholipid FAs may represent 11–32% of total phospholipids-derived side chains in Archaea and 89% in *M. fervidus* [9]. In fact, Archaea have interesting lipids, containing ether linkages, which provide these cells high resilience in environments with high salt concentrations [30,31]. Diether phospholipids in Archaea, which are mirror images of the diester glycerophospholipids found in all other organisms, may also protect them against stereospecific phospholipases excreted by other organisms in competitive environments [30]. In the family *Halobacteriaceae*, the core lipid that is the basis for the most polar lipid structures is 2,3-di-*O*-phytanyl-*sn*-glycerol, with monomethylated phosphatidylglycerophosphate (PGP-Me) being the main phospholipid [32]. The closest species to *Haloarcula* sp. OS, *H. japonica*, *H. marismortui*, and *H. hispanica*, also contain *sn*-2,3-di-*O*-phytanylglycerol exclusively as a core lipid [33,34]. Significant amounts of phospholipid FAs have been previously found in other archaea, including *Halobacterium halobium*, *Methanobacterium thermoautotrophicum*, *Methanococcus jannaschii*, and *Methanopyrus kandleri* [9]. The FA composition of the phospholipids affects the fluidity/rigidity of the cellular membrane in bacteria [35]. Archaeal cells have isoprenoid chains, which are functional over a wide range of temperatures and conditions and, thus, they do not require a regulatory mechanism to adapt lipids during, e.g., environmental temperature changes [36]. However, bacterioruberin is known to increase membrane rigidity in haloarchaea [37].

Accumulating evidence has revealed that oxidative stress and inflammation play a key role in the development and progression of many pathologies, including atopic dermatitis, psoriasis, inflammatory bowel disease, cardiovascular disease, and cancer, among others. It has been shown that in an inflammatory process, immune cells release large amounts of ROS, which induce damage to macromolecules such as DNA, lipids, and proteins, as well as lead to the production of pro-inflammatory mediators [38]. For this reason, the discovery of new agents that attenuate the inflammatory process and oxidative stress can prevent the development of these chronic pathological conditions. Firstly, our results evidenced the antioxidant capacity of the HAE extracted from the new *Haloarcula* sp. OS strain by using ABTS assays. Therefore, the antioxidant activity of the HAE in LPS-stimulated THP-1 macrophages was further evaluated as an in vitro model of oxidative stress. HAE significantly reduced intracellular ROS production induced by LPS, thus protecting cells from oxidative stress. These results are consistent with a variety of studies reporting the antioxidant activity of carotenoids [39,40], and also of plant and microbial extracts [41,42,43,44]. 

Overproduction of ROS results in the release of high levels of pro-inflammatory cytokines such as TNF-α and IL-6. These cytokines can activate immune cells to generate more inflammatory mediators and amplify the immune inflammatory response, leading to tissue damage [45]. In the present study, HAE exerted marked anti-inflammatory activity through the reduction in TNF-α and IL-6 production. Consistent with these findings, there is only one study that evaluates the anti-inflammatory activity of nanoparticles containing a bacterioruberin extract plus dexamethasone. These authors reported that these nanoparticles reduced ROS and TNF-α levels to a higher extent than dexamethasone nanoparticles in a gut inflammation model consisting of Caco-2 cells and LPS-stimulated THP-1 cells [46]. 

Nrf2 is a redox-sensitive transcription factor that under oxidative stress translocates to the nucleus and activates the expression of antioxidant genes such as HO-1. Previous studies have shown that the increase in Nrf2-mediated HO-1 has anti-inflammatory effects through the inhibition of many pro-inflammatory mediators, including TNF-α and IL-6 [47]. It is worth highlighting that the results of our study reported that dexamethasone, a strong and widely used glucocorticoid, decreased ROS production and levels of pro-inflammatory cytokines TNF-α and IL-6 in LPS-stimulated THP-1 macrophages, but its mechanism of action does not appear to be linked to the Nrf/HO-1 signaling pathway. Therefore, HAE involves an antioxidant and anti-inflammatory alternative to classical glucocorticoids such as dexamethasone. Furthermore, LPS-stimulated THP-1 macrophages showed an increase in HO-1 protein expression but not Nrf2 protein expression, which may be explained by an activation of Nrf2 basal cellular levels in response to increased LPS-induced ROS levels. Our results demonstrate that HAE significantly increased the Nrf2 and its main target gene HO-1 protein expression at 50 µg/mL. Additionally, although HAE at 25 µg/mL did not show increased Nrf2 expression, a significant up-regulation of HO-1 expression level was observed, suggesting that HAE may also induce the activation of the Nrf2 basal cellular levels. These findings suggest that up-regulation of the Nrf2/HO-1 signaling pathway may prevent LPS-induced increase in the pro-inflammatory cytokines TNF-α and IL-6. Consistent with these results, a previous study by our group demonstrated that carotenoid fucoxanthin increased the expression of Nrf-2/HO-1 proteins in a UVB-induced erythema model in mice, protecting skin against UV exposure [48]. 

Additionally, it is well known that the bioaccessibility and bioavailability of carotenoids depend on the proportion of these molecules solubilized in micelles that are formed with other lipophilic compounds, such as FAs, cholesterol and phospholipids [49]. In fact, FAs-containing lipids, especially those containing unsaturated bonds, can contribute to scavenger activity and could have a synergic antioxidant effect with carotenoids, improving their bioavailability and harnessing their transport across cell membranes due to their amphipathic nature [49]. In accordance with these observations, we hypothesized that FAs present in the HAE may contribute to the antioxidant activity of the bacterioruberin. 

It is well established that diets rich in polyunsaturated FAs and pigments, as well as the use of these compounds in supplements, have significant health benefits [35]. The HAE contained mainly C18 FAs, including the branched 18:0 10-methyl, and the polyunsaturated 18:2 w6c and 18:3 w6c. Branched-chain FAs, which also include *iso* and *anteiso* FAs, have been found to have a beneficial anti-inflammatory role by increasing the expression of IL-10 [50], as well as 18:2 w6c and 18:3 w6c have been associated to a lower risk of rheumatoid arthritis [51] and anti-inflammatory and antithrombotic properties [52,53].

Based on the results of the present paper, the authors are aware of the limitations of this study but also its potential. In this regard, we have only performed in vitro experimental work with an acetone extract enriched in the carotenoid bacterioruberin and FAs. Therefore, further studies focused on optimizing culture conditions would be of interest to obtain extracts or batches with a similar composition in FAs and bacterioruberin, as well as higher amounts of bacterioruberin *per se*, since this promising carotenoid is poorly investigated. On the other hand, the potential of HAE as an antioxidant/anti-inflammatory agent has been evidenced in the present work, but additional in vivo bioaccessibility and bioavailability studies are necessary to demonstrate its therapeutic potential and explore further mechanistic studies. Numerous studies on the lipid composition, including FAs and carotenoids, of, e.g., microalgae have been published but studies on the composition of Archaea are still scarce [54,55]. Our study suggests that the latter are necessary for future biotechnological applications of Archaea.

## 5. Conclusions

In summary, the results show that the new strain *Haloarcula* sp. OS isolated from Odiel Salworks produces the carotenoid bacterioruberin and significant amounts of FAs. The findings show that the HAE exhibits high antioxidant capacity by the ABTS method. In addition, this study demonstrates, for the first time, that the HAE reduces LPS-induced oxidative stress and acute inflammation in THP-1 macrophages by attenuating both ROS levels and the production of the pro-inflammatory cytokines TNF-α and IL-6. The mechanisms underlying these protective effects are related, at least in part, to the activation of the Nrf-2/HO-1 signaling pathway. Furthermore, the HAE involves an alternative mechanism of action to classical glucocorticoids as dexamethasone. These contributions support the potential use of HAE as a therapeutic agent in the treatment of oxidative stress-related inflammatory diseases.

## Figures and Tables

**Table 1:** Fatty acid composition of the acetone extract of *Haloarcula* sp. OS (HAE) (represented in ng of fatty acids per mg of cell wet weight and as a percentage of total fatty acid content).

FA	ng Lipids/mgCDW	% (FA/Total FAs)
12:0	36.1	2.1
14:0	8.1	0.5
14:0 anteiso	8.9	0.5
16:0	101.7	6.0
16:0 DMA	5.6	0.3
16:0 N alcohol	62.5	3.7
17:0	7.2	0.4
18:0	948.2	55.5
18:0 10-methyl	230.7	13.5
18:1 w9c	95.1	5.6
18:2 w6c	16.0	0.9
18:3 w6c	187.4	11.0

**Table 2:** Viability of THP-1 human macrophages treated with different concentrations of the *Haloarcula* sp. OS acetone extract (HAE) at different concentrations. Values are the means ± SEM (%), (N = 3).

	% Viability THP-1 Macrophages (24 h)					
(µg/mL)	0	6.25	12.5	25	50	100
HAE	100 ± 2.2	112.5 ± 6.5	111.40 ± 4.70	117.06 ± 5.5	111.60 ± 9.7	114.51 ± 11.5

## Data Availability

Not applicable.

## Supplementary Materials

The following supporting information can be downloaded at: https://www.mdpi.com/article/10.3390/antiox12051080/s1, Figure S1: Growth curve of the isolated haloarchaea *Haloarcula* sp. OS (HAE); Figure S2: Full length of the 16S rRNA encoding gene from *Haloarcula* sp. OS, amplified with the archaeal-specific primers 21F (5′-TTCCGGTTGATCCTGCCGGA-3′) and 1492R (5′-GGTTACCTTGTTACGACTT-3′).
