# Supplementary material for: Up-Regulation of the Nrf2/HO-1 Antioxidant Pathway in Macrophages by an Extract from a New Halophilic Archaea Isolated in Odiel Saltworks
Source: Antioxidants (Basel). 2023 May 11;12(5):1080. doi: 10.3390/antiox12051080 (PMC10215637; doi:10.3390/antiox12051080)
Supplement: Supplementary file 1 [file antioxidants-12-01080-s001.zip › Suplementary Mat_HAE.pdf]

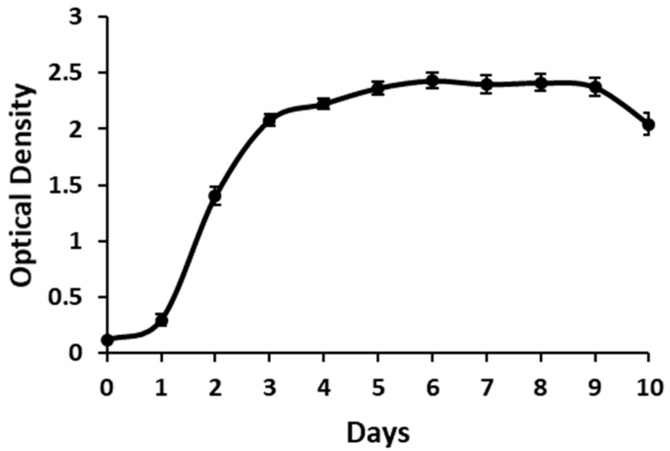

Supplementary Figure S1. Growth curve of the isolated haloarchaea *Haloarcula* sp. OS. The optical density was measured every day at 580 nm. This microorganism showed fast growth (μ=1.02 days^{-1}). The replicates were obtained from two cultures in parallel, started with an inoculum at the end of the exponential phase and the OD was measured in 1 mL cuvettes.

CGATTAGCCCTGCTAGTCGCACGGGTCTTAGACTCCGTAGGC
ATATAGCTCAGTAACACGTGGCCAAACTACCCTACAGACCGC
GATAACCTCGGGAAACTGAGGCCAATAGCGGATATAACTCTC
AGGCTGGAGTGCCGAGAGTTAGAAACGTTCCGGCGCTGTAGG
ATGTGGCTGCGGCCGATTAGGTAGATGGTGGGGTAACGGCCC
ACCATGCCGATAATCGGTACGGGTTGTTGGAGAGCAAGAACC
CGGAGACGGTATTTGAGACAAGATACCGGGCCCTACGGGGC
GCAGCAGGCGGGAAACCTTTACACTGCACGACAGTGCGATA
GGGGGACTCCGAGTGTGAGGGCATATAGCCCTCGCTTTTCTG
TACCGTAAGGTGGTACAGGAACAAGGACTGGGCAAGACCGG
TGCCAGCCGCCGCGGTAATACCGGCAGTCCAAGTGATGGCCG
ATATTATTGGGCCTAAAGCGTCCGTATTCGGCCGGACAAGTC
CGTTGGGAAATCGACGAGCTCAACTGGTCGGCGTCCAGTGGA
AACTACCCGGCTTGGGGCCGGAAGACTTGACGGGTACGTCCG
GGGTAGGAGTGAAATCCTGTAATCCTGGACGGACCACCAATG
GGGAAACCACCTCAGGAAGCCGGACCCGACGGTGAGGGACG
AAAGCCAGGGTCTCGAACCGGATTAGATACCCGGGTAGTCCT
GGCTGTAAACGATGCTCGCTAGGTGTGCCGTAGGCCACGAGC
ATGCGATGCGCCGTAGTGAAGCCGAGAAGCGAGCCGCCTGG
GAAGTACGTCCGCAAGGATGAAACTTAAAGGAATTGGCGGG
GGAGCACCACAACCGGAGGAGCCTGCGGTTTAATTGGACTCA
ACGCCGGAAATCTCACCGGTCCCGACAGTAGTAATGACGGTC
AGGTTGACGACTTTACTCGACGCTACTGAGAGGAGGTGCATG
GCCGCCGTCAGCTCGTACCGTGAGGCGTCCTGTTAAGTCAGG
CAACGAGCGAGACCCGCACTTCTAGTTGCCAGCAATACCCTT
GAGGTAGTTGGGTACACTAGGAGGACTGCCGCTGCTAAAGCG
GAGGAAGGAACGGGCAACGGTAGGTCAGTATGCCCCGAATG
GACCGGGCAACACGCGGGCTACAATGGCTCTGACAGTGGGA
TGCAACGCCGAGAGGCGACGCTAATCTCCAAACGGAGTCGT
AGTTCGGATTGCGGGCTGAAACCCGCCCGCATGAAGCTGGAT
TCGGTAGTAATCGCGTGTCAGAAGCGCGCGGTGAATACGTCC
CTGCTCCTTGCACACACCGCCCGTCAAAGCACCCGAGTGGGG
TCCGGATGAGGCCGTCATGCGACGGTCGAATC

Supplementary Figure S2. Full length of the 16S rRNA encoding gene from *Haloarcula* sp. OS, amplified with the archaeal specific primers 21F (5′-TTCCGGTTGATCCTGCCGGA-3′) and 1492R (5′-GGTTACCTTGTTACGACTT-3′). Polymerase chain reactions (PCR) were performed as indicated in the Materials and Methods section
